# Effect of Targeted Probiotics on Anesthesia‐Induced Sleep Disturbances via Modulating the Gut Microbiome and Metabolites

**DOI:** 10.1002/fsn3.71447

**Published:** 2026-01-14

**Authors:** Rui‐zhi Yang, Song Lin, Le‐tong Huang, Jing Weng, Qiao‐ming Liu, Han‐shen Chen, Ning Ruan, Kai Zeng

**Affiliations:** ^1^ Department of Anesthesiology The First Affiliated Hospital of Fujian Medical University Fuzhou China; ^2^ National Regional Medical Center, Binhai Campus of the First Affiliated Hospital Fujian Medical University Fuzhou China; ^3^ Department of Anesthesiology, Xiamen Cardiovascular Hospital of Xiamen University, School of Medicine Xiamen University Xiamen China; ^4^ Department of Gastrointestinal Surgery, the First Affiliated Hospital Fujian Medical University Fuzhou China

**Keywords:** anesthesia, gut microbiome, lung microbiome, metabolites, probiotics, sleep

## Abstract

Post‐operative/post‐anesthesia sleep disturbances are a major concern to patients, impacting recovery and overall well‐being. Probiotics may offer potential benefits for sleep promotion by modulating microbial diversity and abundance. This study aimed to investigate the effect of targeted probiotic treatment on anesthesia‐induced sleep disturbances and its impact on the microbiota and metabolites in the gut and lungs. Eight‐week‐old male SD rats received a continuous inhalation of isoflurane, combined with oral yogurt treatment without or containing probiotic *Lactobacillus* and *Bifidobacteria*. Rats underwent electrode implantation and 7 days of polysomnography. 16S rRNA sequencing and untargeted metabolomic analysis from fecal and BALF samples were used to investigate the changes in the gut and lung microbiota and their metabolites. Isoflurane exposure led to sleep disturbances associated with a significant reduction in *Lactobacillus* and *Bifidobacteria* in the gut. Targeted probiotic supplementation improved post‐anesthesia sleep quality (NREM sleep time on day 1: Yogurt+ISO group 597.25 ± 100.15 vs. Probiotic+ISO group 772.77 ± 29.36 min, *p* = 0.002), increased the abundance of beneficial gut bacteria, and reduced wake‐related metabolites in both the gut and lungs. Correlation analysis revealed significant negative correlations between the abundance of beneficial gut flora and wake‐related metabolites (all *p* < 0.05). The present study first indicated that targeted probiotic treatment alleviated post‐anesthesia sleep disturbances by modulating both the gut and lung microbiota and their metabolites. These findings suggest that peri‐anesthesia probiotic treatment may be a viable strategy for improving sleep disturbances, although further clinical research into the underlying mechanisms is needed.

## Graphical abstract

The diversity and abundance of the gut microbiome in rats were significantly disrupted following post‐anesthesia sleep disturbance. *Lactobacillales* and *Bifidobacterium* yogurt supplementations increased sleep quality considerably and the abundance of beneficial bacteria, while reducing wakefulness and wake‐related metabolites in the gut.

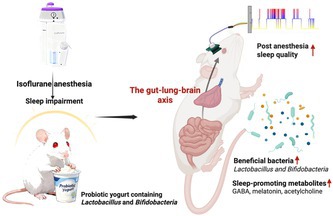

## 1 Introduction

Quality sleep is essential for physical and mental health, and is beneficial for cognition, memory consolidation, immunity, and hormone regulation (Baranwal et al. 2023). Around 60% of surgical patients experience pre‐ and post‐operative sleep disturbances, including inadequate sleep duration, poor sleep quality, and high sleep latency (Butris et al. 2023). In the hospital setting, factors contributing to poor sleep include pre‐existing sleep disturbance and anxiety, older age, post‐operative pain, surgery, isoflurane anesthesia, and ward environment (Jia et al. 2024; Hillman 2021; Joyce et al. 2024). Post‐operative/post‐anesthesia sleep disturbances could lead to cognitive impairment, acute and chronic post‐operative pain, delirium, and delayed recovery (Song et al. 2018; Varallo et al. 2022; O'Gara et al. 2021; Leung et al. 2023). However, there are limited studies investigating safe and effective prophylactic agents for post‐anesthesia sleep disturbances.

Growing evidence has revealed that the gut microbiome is essential for the maintenance of normal sleep physiology through the gut‐brain axis (Wang et al. 2021; Morais et al. 2021). Gut metabolites further regulate the expression of clock genes in the host (Shimizu et al. 2023) and the synthesis of sleep‐promoting signals, especially the short‐chain fatty acids (SCFAs), acetylcholine, and γ‐aminobutyric acid (GABA) (Han et al. 2022). However, accumulated studies have revealed that anesthesia/surgery induced unfavorable changes in the gut microbiome and metabolites (Lian et al. 2021; Serbanescu et al. 2019), even altering the diversity and abundance of lung microbiota (Yang, Liang, et al. 2023).

The lung harbors a distinct microbial community that interacts with the gut microbiota via the gut‐lung axis. This crosstalk occurs through multiple pathways, including bacterial‐derived components, metabolic degradation products, and inflammatory signaling (Dang and Marsland 2019). Furthermore, lung microbiota and their metabolites can communicate directly with the central nervous system (CNS) via the lung‐brain axis (Azzoni and Marsland 2022). Such lung microbial dysbiosis, driven by anesthesia‐induced changes, may in turn affect sleep‐related pathways either independently or synergistically with gut microbial dysbiosis. Therefore, microbiome‐targeted interventions gradually serve as therapeutic measures to ameliorate sleep disturbances induced by microbiome disturbance.

Probiotics are live microorganisms intended to offer health benefits when consumed, primarily by modifying the gut microbiome, which is known to enhance sleep quality (Irwin et al. 2020). Recent clinical studies have demonstrated the efficacy and safety of multi‐strain probiotic mixtures for improving sleep, especially the most studied *Lactobacillus* and *Bifidobacteria* (Lee et al. 2021; Rode et al. 2022). However, to date, no studies have yet simultaneously analyzed gut and lung metagenomics and metabolomics in the same subjects to investigate the effect of targeted probiotic treatment on sleep improvement following anesthesia, nor to elucidate the interactions between the gut and lung microbiome and their metabolites.

## 2 Materials and Methods

### 2.1 Animals

Experiments were approved by the Ethics Committee of Fujian Medical University on 8 October 2023 (Approval number: IACUC FJMU 2023‐0260). SPF‐grade male SD rats were used in all experiments (8 weeks old). Rats were maintained under SPF conditions at a constant temperature, with food and water given ad libitum. After being randomly assigned to different groups using computer‐generated random numbers, the different groups of rats were raised in separate cages to prevent cross‐contamination of microbiota between the groups.

### 2.2 Study Protocol

Rats were randomly divided into (1) control group (no intervention), isoflurane group (inhalation of isoflurane); (2) FMT‐CON group (pseudo‐germfree rats receiving fecal microbiota transplantation from control rats) and FMT‐ISO group (pseudo‐germfree rats receiving fecal microbiota transplantation from isoflurane‐anesthesia rats); (3) Yogurt+ISO group (inhalation of isoflurane and pretreatment with probiotic‐free yogurt) and Probiotic+ISO group (inhalation of isoflurane and pretreatment with probiotic yogurt). In the isoflurane group, rats were induced in an induction chamber with 3% isoflurane. After losing the righting reflex, they were adjusted to a maintenance level of 1.5% isoflurane for 4 h. We used an isoflurane‐specific calibrated vaporizer to deliver 50% oxygen at 3 L/min (Yang, Liang, et al. 2023). Next, the rats were administered compound antibiotics to eliminate intestinal microbiota and subsequently received FMT from control or isoflurane‐anesthesia rats to investigate the link between sleep disturbances and gut microbiome dysbiosis caused by anesthesia. Following this, the rats were pretreated with probiotic or probiotic‐free yogurt for 14 days to modulate microbiota dysbiosis. Finally, fecal samples and bronchoalveolar lavage fluids (BALFs) were collected for high‐throughput 16S rRNA sequencing. We used polysomnography to test the sleep quality of rats.

### 2.3 Microbiota Transplantation

To build the pseudo‐germfree rat model, rats in the FMT group were administered an antibiotic cocktail containing 1 mg mL^−1^ ampicillin sodium, 1 mg mL^−1^ neomycin sulfate, 1 mg mL^−1^ metronidazole, and 0.5 mg mL^−1^ vancomycin hydrochloride in drinking water for 14 consecutive days to deplete the microbiota (Han et al. 2021). Intestinal microbiota donors were isoflurane‐anesthetized or control rats, with fresh fecal samples prepared on the day of transplantation and administered within 3 h. Following antibiotic treatment, the rats received 200 μL fecal supernatant via gastric administration daily for 1 week. After the FMT, we performed a one‐week polysomnography.

### 2.4 Surgery for Electrode Implantation and EEG/EMG Recordings and Analysis

To monitor sleep activity in freely moving rats, recordings were obtained using custom electrodes containing EEG and EMG. Rats were anesthetized with 50 mg/Kg sodium pentobarbital, and eye ointment was applied to both eyes. Four EEG electrodes were implanted: two over the frontal cortical area (A/P = +1.5 mm; M/L = ±1.5 mm), and two over the parietal area (A/P = −3.5 mm; M/L = ±1.5 mm) and served as recording electrodes. The EMG leads were fixed to the neck muscles of rat to serve as reference electrodes for noise reduction. EEG and EMG signals were recorded with a TDT RZ5 amplifier, filtered (1–300 Hz) and digitized at 1000 Hz in Medusa Server (Bio‐Signal) (Dong et al. 2022). Rats were housed in a single cage for a week.

The animals were housed individually in an EEG/EMG recording cage under a 12‐h light: 12‐h dark cycle (with light onset at 07:00 and offset at 19:00) for a week before polygraphic recording. Automated analysis categorized EEG/EMG signals into wake, non‐rapid eye movement (NREM) sleep, and rapid eye movement (REM) sleep states every 4 s using the Lunion Stage AI engine (LunionData, Shanghai, China), with manual verification and correction. EEG signals were filtered between 0.3 and 35 Hz, and EMG signals between 10 and 200 Hz (Zhang et al. 2024). Interference was considered when the recorded waves did not fit the patterns determined for any sleep parameter.

### 2.5 Accurate 16S Absolute Quantification Sequencing and Analysis

Microbial DNA was extracted using the TransStart TopTaq DNA Polymerase kit (Transgen, China) following the manufacturer's instructions. Afterwards, Primer F (Illumina adapter sequence 1+ CCTACGGGNGGCWGCAG) and Primer R (Illumina adapter sequence 2+ GACTACHVGGGTATCTAATCC) were used to generate amplicons from the V3–V4 regions of the 16S rRNA gene. To ensure precise and efficient amplification, the spike‐in inner reference DNA and sample DNA (10 ng/μL) were utilized along with a positive control. Subsequently, Illumina 2 × 250 bp paired‐end sequencing was carried out, followed by bioinformatics analysis, including operational taxonomic units (OTUs) analysis, community composition analysis, alpha diversity analysis, and beta diversity analysis. Statistical analysis was conducted in R (v3.5.1).

### 2.6 Untargeted Metabolomic Analysis

The feces and BALF samples were prepared for metabolomic analysis according to the manufacturer's instructions (Genesky Biotechnologies Inc., Shanghai). An ultra‐performance liquid chromatography–tandem mass spectrometry (LC–MS/MS) system was used for analysis. LC analysis was performed on a Vanquish UHPLC System (Thermo Fisher Scientific, USA). Chromatography was conducted on ACQUITY UPLC HSS T3 (2.1 × 100 mm, 1.8 μm; Waters, Milford, MA, USA). Metabolites were detected by MS on Thermo Orbitrap Exploris 120 (Thermo Fisher Scientific, USA) equipped with an ESI ion source. Software R (R version R‐3.4.3) and Python (Python 2.7.6 version) were utilized for statistical analyses. Kyoto Encyclopedia of Genes and Genomes (KEGG) database (https://www.genome.jp/kegg/pathway.html), HMDB database (https://hmdb.ca/metabolites), and LIPIDMaps database (http://www.lipidmaps.org/) were used for metabolites classification annotation.

### 2.7 Statistical Analysis

The data analysts were independent of the experimental procedures and did not participate in the implementation of the experiments. Sample size was determined based on the wakefulness levels in the control and Isoflurane groups, using an Effect Size (Cohen's *d*) Calculator and the R package “pwr”. Cohen's *d* = (Mean1 − Mean2)/√[(Standard Deviation1^2^) + (Standard Deviation2^2^)/2]. The calculated minimum sample size required was 4. To account for potential dropouts, a final sample size of *n* = 5 was selected for each group.

Data were expressed as means ± standard deviations (SD) using GraphPad (version 9.5.0, GraphPad Software Inc., La Jolla, CA, USA), and evaluated by a one‐way analysis of variance (ANOVA). Spearman correlation analysis was employed to investigate the correlation between the abundance of microbiota and metabolites. Statistical significance was set at *p* < 0.05.

## 3 Results

### 3.1 Isoflurane Anesthesia Altered the Gut Microbiota of Rats More Than the Lung Microbiota

**FIGURE 1:**
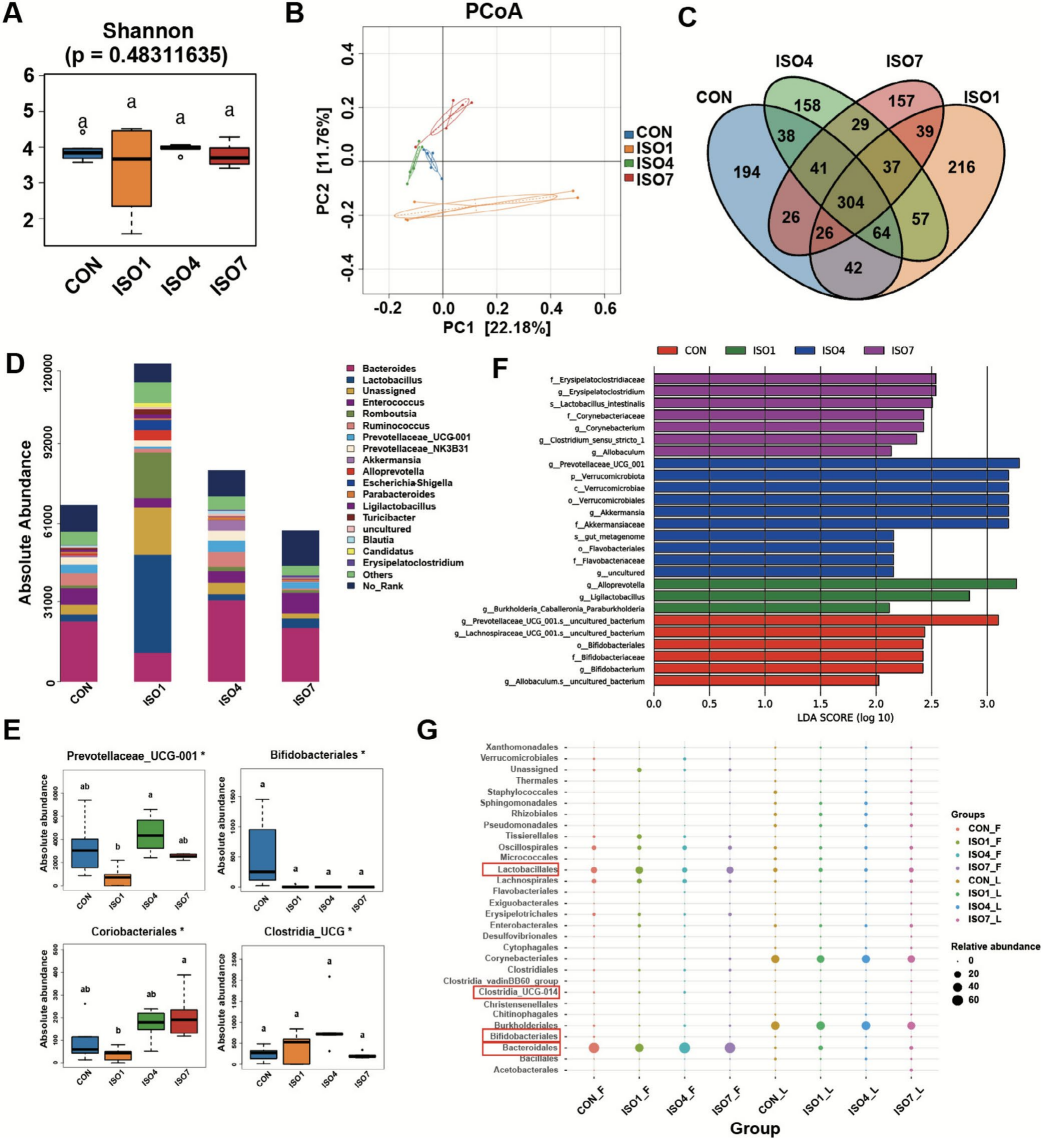
Isoflurane anesthesia altered the gut microbiome of rats. (A) Analysis of Alpha diversity (Shannon diversity index). (B) In the analysis of Beta diversity (PCoA), the dispersed distribution positions of each group indicate their differences. (Different colors represent different groups, and the further the distance, the greater the difference in microbial communities.) (C) OTU Venn diagram showed that 304 OTUs were shared by all groups, and the difference between each group was evident. (D) At the genus level, the absolute abundance of Bacteroides, Enterococcus, Lactobacillus, Prevotellace‐ae_UCG‐001, and Romboutsia statistically changed after isoflurane inhalation. (Different colors correspond to different species, and the color block length indicates the absolute abundance of the species represented by the color.) (E) At the genus level, the absolute abundance of Bifidobacterium and Clostridia_UCG‐014 decreased significantly in isoflurane‐inhaled rats, especially on the first day. (G) In the combined analysis of the gut (F) and lung (L) microbiome, Bacteroidales, Lactobacillales, Clostridia_UCG‐014, and Bifidobacterium were the most relevant differential floras after isoflurane anesthesia. *n* = 5 for each timepoint. “ISO1” represents the first day post isoflurane exposure, “ISO4” indicates the fourth day, and “ISO7” signifies the seventh day.

We performed 16S rRNA sequencing on fecal and BALF samples from rats to investigate the impact of isoflurane on the diversity and abundance of microbiota. Analysis of alpha diversity (Shannon index) revealed no significant differences in the gut microbiota diversity between the control and isoflurane‐exposed groups (Figure 1A). However, beta diversity via the PCoA analysis showed a dispersed distribution that presented a significant difference among the groups (Figure 1B). As shown in the Venn diagram, 304 OTUs were shared by all groups, and the difference between each group was evident (Figure 1C). At the genus level, the absolute abundance of the gut microbiome statistically increased after isoflurane inhalation and then recovered to control levels on day 7. The top five most variable strains are *Bacteroides, Enterococcus, Lactobacillus, Prevotellaceae_UCG‐001*, and *Ruminococcus*. Notably, the absolute abundance of *Bifidobacterium* and *Clostridia_UCG‐014* decreased significantly in isoflurane‐exposed rats, especially on the first day (Figure 1D,E). Differential species identified by LDA were consistent with these findings. Beneficial microorganisms such as *Bifidobacterium* and *Clostridia_UCG‐014* were more abundant in the control group, while *Akkermansia* was significantly reduced after anesthesia (Figure 1F). LDA > 2 and *p* < 0.05 were considered significant.

However, isoflurane anesthesia appeared to have a minimal effect on the lung microbiota in rats. Figure S1 showed no statistical differences in the alpha or beta diversity between the control and isoflurane groups. We also classified the OTUs present in these groups. At the genus level, the absolute abundance of bacteria producing succinic acid, such as *Bacteroidales*, increased in isoflurane‐inhaled rats, whereas beneficial bacteria *Lactobacillales* decreased, although this change was not statistically significant.

In the combined analysis of the gut and lung microbiome, we observed that *Bacteroidales, Lactobacillales, Clostridia_UCG‐014*, and *Bifidobacterium* exhibited a similar change trend before and after isoflurane intervention, highlighting these as the most relevant differential floras associated with isoflurane anesthesia (Figure 1G). Collectively, these data indicated that isoflurane anesthesia significantly altered the gut microbiota of rats.

### 3.2 Isoflurane Anesthesia Impaired Sleep in Rats

**FIGURE 2:**
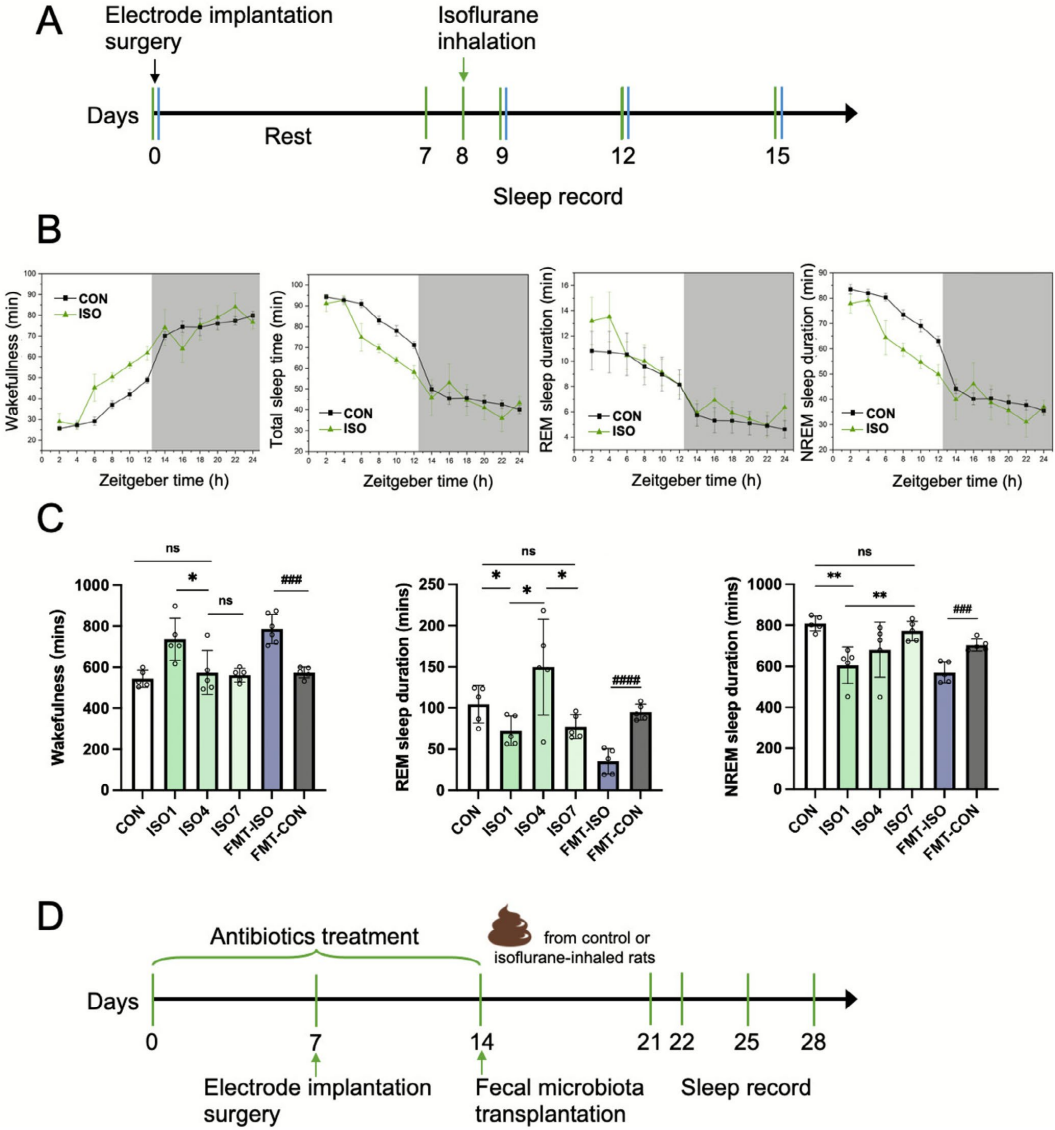
Isoflurane anesthesia impaired sleep in rats. (A) Experimental timeline of the control and isoflurane anesthesia rats. (B) Comparison of cumulative time spent in wakefulness, total sleep time, NREM, and REM sleep duration between the control and isoflurane groups (ISO4) during the light and dark (gray area) periods over 24 h. (C) Sleep record, including wakefulness, REM, and NREM sleep duration. (D) Experimental timeline of the fecal microbiota transplantation rats. *n* = 5 for each group. Statistical analysis was done using two‐way ANOVA followed by Tukey post‐test. *p < 0.05, **p < 0.001 versus control; ^*###*^ *p* < 0.001, ^*###*^ *p* < 0.0001 versus FMT‐CON.

To address whether anesthesia affected sleep in rats, we used polysomnography to assess sleep quality following isoflurane anesthesia. The sleep record was conducted on days 1, 4, and 7 post‐anesthesia (Figure 2A). The time spent in each sleep–wake stage across the light and dark period at a scale of 2‐h interval was demonstrated in Figure 2B. We found significant differences between the control and isoflurane groups, particularly on day 1 following isoflurane exposure (Figure 2C). Wakefulness increased significantly on day 1 after isoflurane anesthesia compared to the baseline (*p* = 0.005), and subsequently began to return to the baseline level by day 4. REM sleep duration differed over time; rats anesthetized with isoflurane showed significantly reduced REM sleep time compared to the control group, except on day 4 (*p* = 0.076). Isoflurane significantly impaired NREM sleep after anesthesia since day 1 (*p* = 0.002), then recovering to normal levels by day 7.

Given that isoflurane anesthesia impacted the gut microbiota more than the lung microbiota in rats, FMT experiments were performed to explore the causal relationship between anesthesia‐induced sleep changes and the gut microbiota (Figure 2D). After FMT from rats that experienced sleep impairment caused by isoflurane, recipient rats also exhibited sleep disorders (Figure 2C). Compared to the FMT‐CON group receiving normal fecal transplant, these recipients showed a significant increase in wakefulness and notable reductions in both REM and NREM sleep duration (all *p* < 0.001).

### 3.3 Pretreatment With Probiotic Yogurt Alleviated Anesthesia‐Induced Sleep Disturbances

**FIGURE 3:**
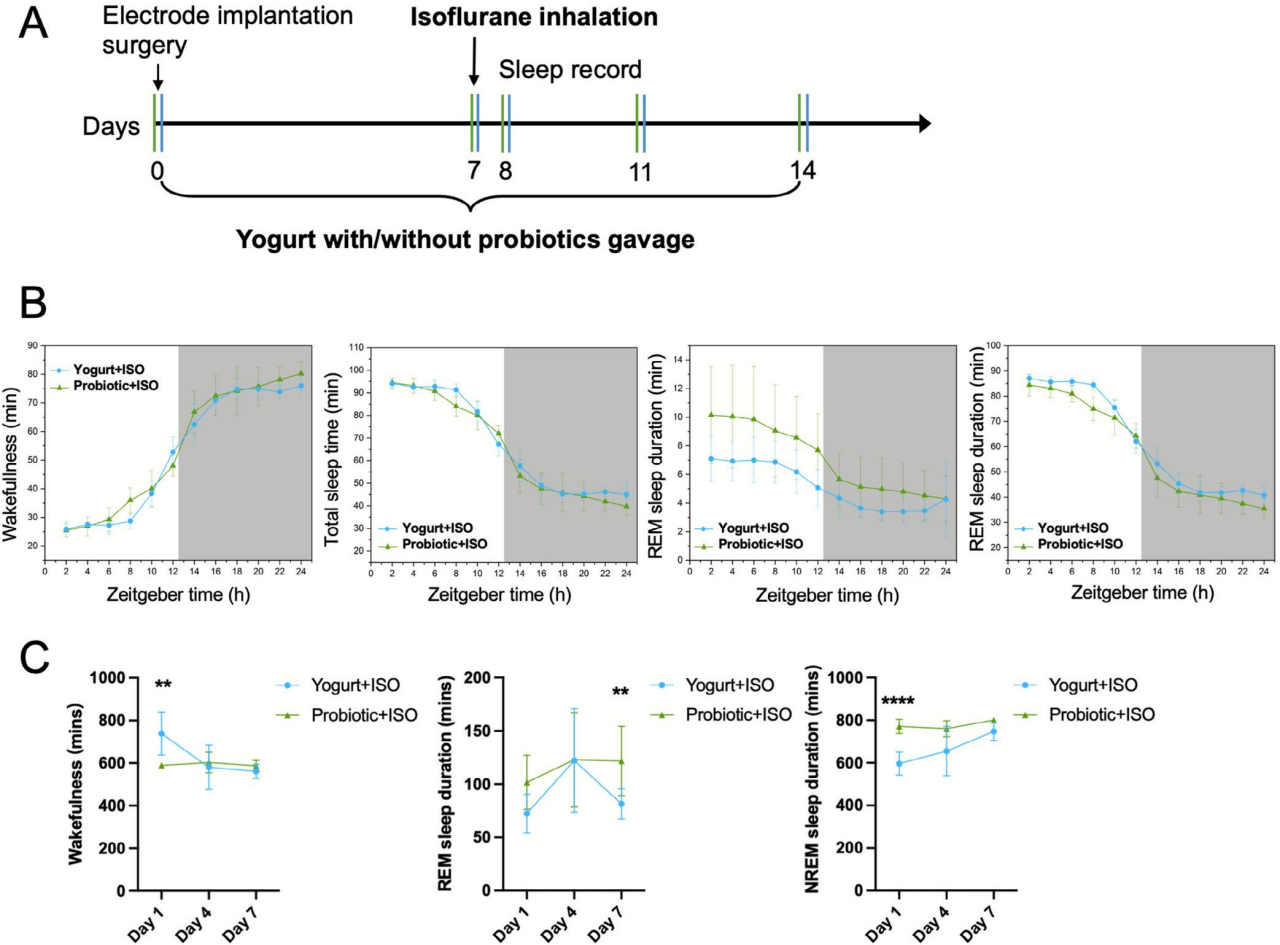
Probiotic yogurt improved sleep quality in isoflurane anesthesia rats. (A) Experimental timeline of the yogurt with or without probiotics combined with isoflurane anesthesia rats. (B) Comparison of cumulative time spent in wakefulness, total sleep time, NREM, and REM sleep during the light and dark (gray area) periods over 24 h between the yogurt+isoflurane and probiotic+isoflurane groups on post‐anesthesia day 7. (C) Sleep records, including wake, REM, and NREM sleep time. *n* = 5 for each group. Statistical analysis was done using two‐way ANOVA. **p < 0.01, ****p < 0.0001, versus Yogurt+ISO group.

According to the differential flora of the gut and lung microbiota between the control and isoflurane groups, we chose the fermented yogurt containing *Bifidobacterium animalis subsp., Streptococcus thermophilus subsp*., and *Lactobacillus delbrueckii subsp*. for gavage administration for 14 days to correct the microbiota dysbiosis. A negative control group was administered yogurt without probiotics. Sleep recordings were conducted on days 1, 4, and 7 after anesthesia (Figure 3A). Probiotic yogurt treatment mitigated the negative effects of isoflurane inhalation compared to the no‐probiotic group. Rats in the Probiotic+ISO group showed significantly reduced wakefulness on day 1 (*p* = 0.008), increased REM sleep on day 7 (*p* = 0.002), and enhanced NREM sleep on day 1 (*p* < 0.001). These effects were statistically different compared to those observed in the Yogurt+ISO group, underscoring the effectiveness of probiotic treatment (Figure 3B,C).

### 3.4 Probiotic Yogurt Changed the Diversity and Abundance of the Gut and Lung Microbiota and Metabolites

**FIGURE 4:**
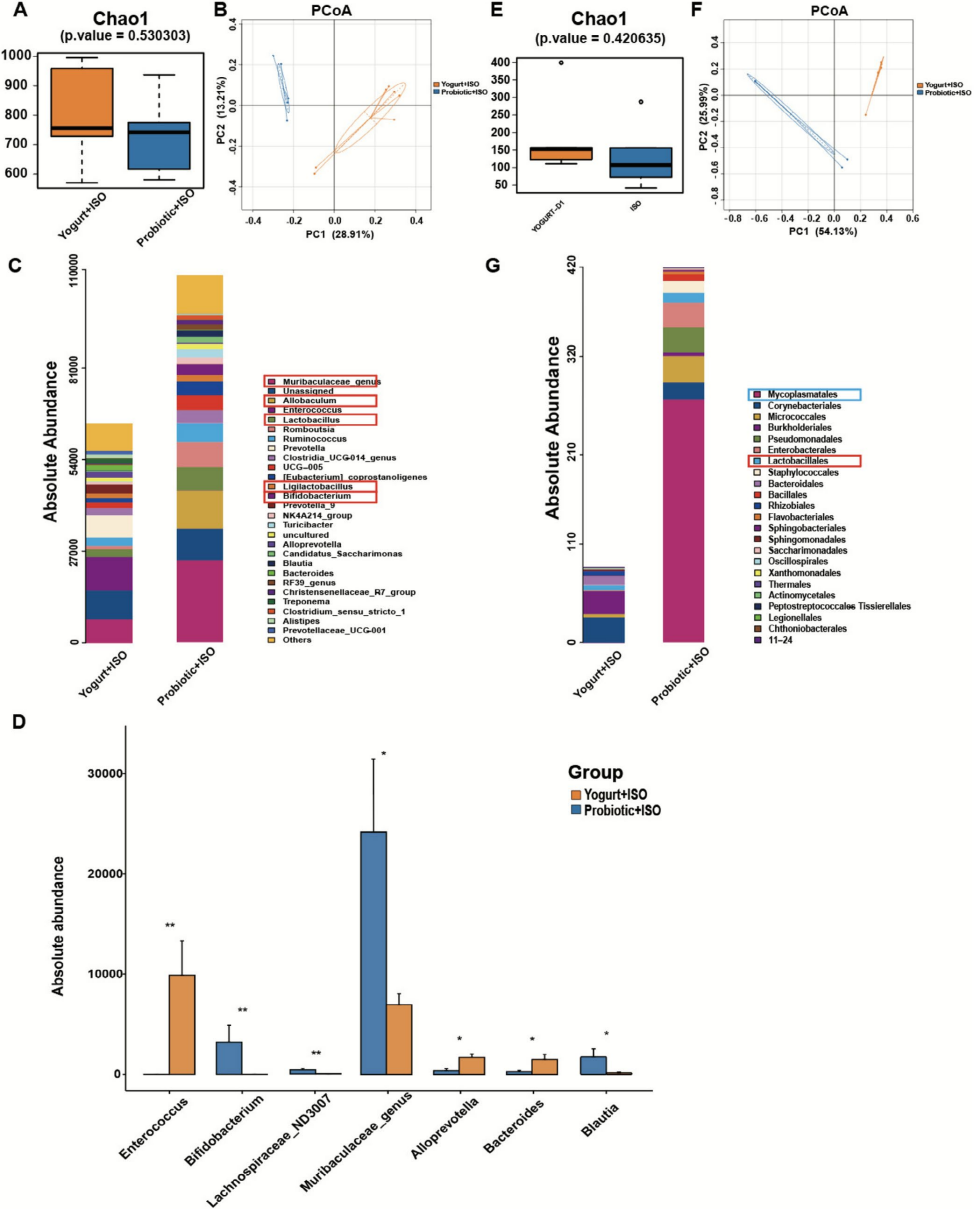
Probiotic yogurt altered the diversity and abundance of the gut and lung microbiota. (A) The alpha diversity (Chao1 index) of gut microbiota. (B) Scatter plots of PCoA for microbial composition in cecal contents. (C) Microbiota composition and absolute abundance at genus levels in the cecum of rats. (D) At the genus level, there was an increase in the abundance of beneficial flora (*Muribaculaceae_genus*, *Allobaculum*, *Lactobacillus*, *Ligilactobacillus*, and *Bifidobacterium*). (E) The alpha diversity (Chao1 index) of lung microbiota. (F) Scatter plots of PCoA for lung microbiota. (G) Composition at genus levels in the lung microbiota. Only *Mycoplasmatales* in the Probiotic+ISO group significantly increased in abundance. *n* = 5–7 for each group. Data are expressed as mean ± SD. *p < 0.05, **p < 0.01.

16S rRNA sequencing revealed significant changes in the gut microbiota diversity and abundance following probiotic yogurt treatment. The alpha diversity measured by the Shannon index in the gut microbiota of the Yogurt+ISO group was significantly higher than that in the Probiotic+ISO group (*p* = 0.004, Figure 4A). The results of PCoA in beta diversity indicated significant differences in the distribution of gut microbiota among groups, with distinct independent clusters (Figure 4B). At the phylum level, *Firmicutes*, *Bacteroidetes*, and *Actinobacteria* were the predominant populations in both groups. At the genus level, probiotics notably increased the absolute abundance of beneficial flora, with the top 5 genera being *Muribaculaceae_genus*, *Allobaculum*, *Lactobacillus*, *Ligilactobacillus*, and *Bifidobacterium*. Conversely, the abundance of *Enterococcus* decreased significantly (Figure 4C,D).

16S rRNA sequencing was performed on the lung microbiome for both groups simultaneously. Although the Chao1 index did not differ between the groups, PCoA results showed significant differences in the distribution of the lung microbiome, with the two groups clustering into distinct and separate clusters (Figure 4E,F). At the genus level, there was a significant increase in the abundance of *Mycoplasmatales* in the Probiotic+ISO group. *Bifidobacterium* levels also increased, but this change was not statistically significant (Figure 4G).

**FIGURE 5:**
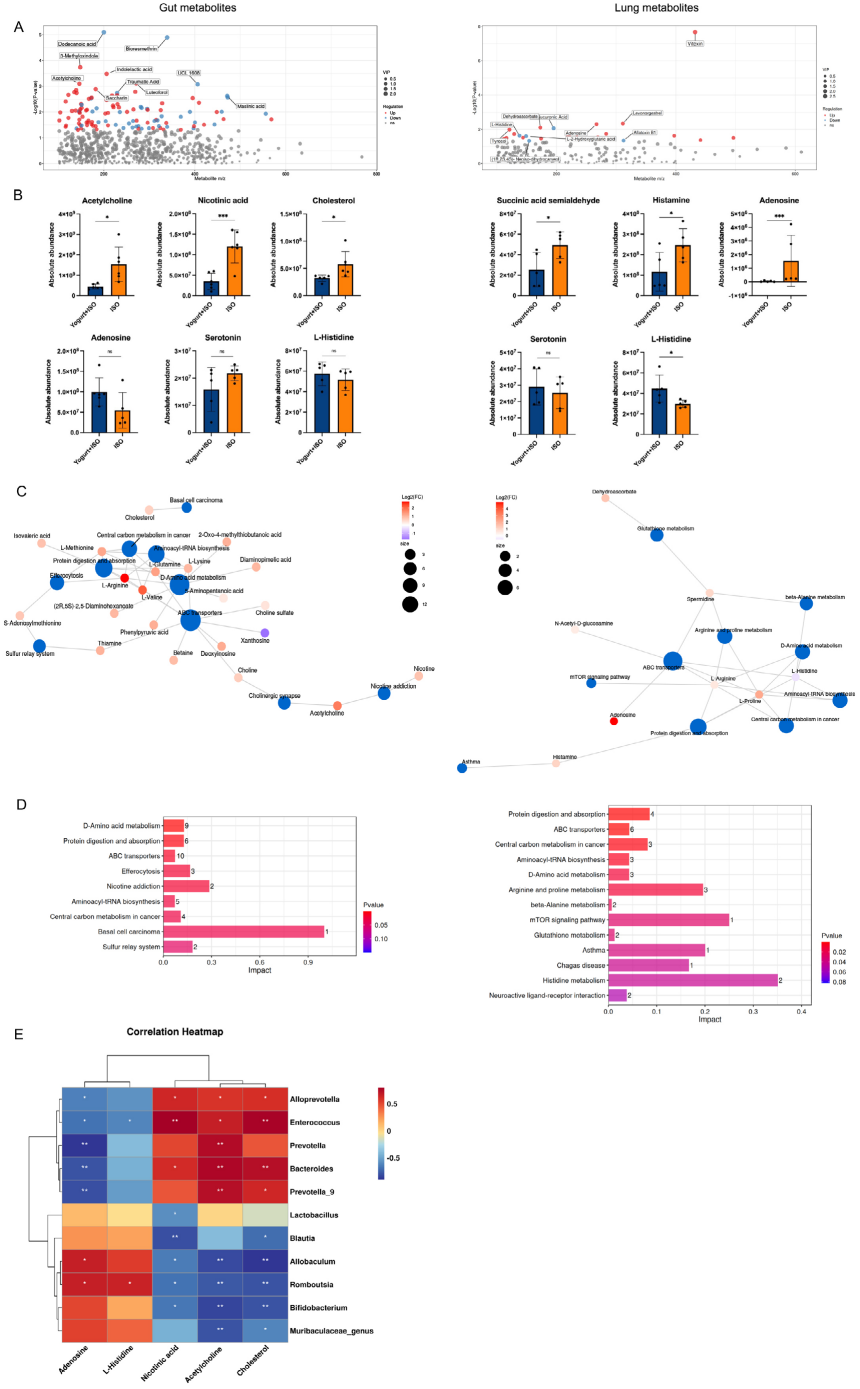
Changes in the gut and lung metabolites. (A) Volcanic map of differential metabolites. Each point represents a metabolite, and the ordinate represents the ‐log10 value of the *p*‐value. The larger the ordinate value, the more significant the differentially expressed metabolites (Red dots and blue dots represent up‐regulated and down‐regulated differentially expressed metabolites, respectively). (B) At the genus level, the absolute abundance of metabolites is associated with sleep in the gut and lungs. (C) Network diagram of KEGG enrichment analysis. Dark blue dots indicate pathways and other color dots indicate differential metabolites. The larger the pathway point, the greater the number of differential metabolites associated with it. The metabolite point indicates the magnitude of the log2 (FC) value by gradients. (D) Metabolic pathway influence factor bar chart of KEGG enrichment analysis. The horizontal coordinate represents the Impact value that is enriched into different metabolic pathways. The higher the value, the more the contribution of differential metabolites detected in this pathway. (E) Correlation analysis of differential microbiome with metabolites. *n* = 5–7 for each group. Data are expressed as mean ± SD. *p < 0.05, ***p < 0.001.

To screen potential metabolic targets that might be linked with post‐anesthesia sleep disturbances, untargeted metabolomics profiling of gut and lung metabolites was performed. A total of 73 positive and 40 negative differential gut metabolites were identified in Yogurt+ISO versus Probiotic+ISO groups. Notably, key metabolites with significant differences included dodecanoic acid, 3‐Methyloxindole, indolelactic acid, and acetylcholine between the groups (Figure 5A). Compared with the no‐probiotic group, the probiotic yogurt significantly decreased the absolute abundance of acetylcholine, nicotinic acid, and cholesterol (all *p* < 0.05), with a slight but non‐significant decrease in serotonin and an increase in L‐Histidine in the Probiotic+ISO group (Figure 5B). Adenosine levels did not differ significantly between the groups. Regarding lung metabolites, 20 positive and 5 negative differential metabolites were identified. Among these, vitexin, levonorgestrel, adenosine, dehydroascorbate, and D‐glucuronic acid exhibited the most pronounced differences (Figure 5A). Among the differential metabolites, succinic acid semialdehyde, histamine, and adenosine were significantly enriched in the Yogurt+ISO group. Additionally, L‐Histidine (*p* = 0.041) and serotonin (*p* = 0.597) showed increased absolute abundance following probiotic yogurt supplementation (Figure 5B).

The top enriched KEGG pathways of differential metabolites between the Yogurt+ISO and Probiotic+ISO groups were illustrated in Figure 5C,D. In gut metabolites, significant pathway enrichment included D‐Amino acid metabolism, Protein digestion and absorption, ABC transporters, and Aminoacyl‐tRNA biosynthesis, with key metabolites enriched in L‐Arginine and L‐Valine. Similarly, lung metabolites showed enrichment in pathways such as Protein digestion and absorption, ABC transporters, Aminoacyl‐tRNA biosynthesis, D‐Amino acid metabolism, and Neuroactive ligand‐receptor interaction, with metabolites primarily enriched in adenosine. Overall, these pathways reflect broader themes of sugar and energy metabolism, amino acid metabolism, antioxidant and repair mechanisms, vitamin and coenzyme metabolism, transfer systems, and more. Their influence on sleep quality may be mediated through effects on cellular function, neurotransmitter balance, antioxidant defense, and other physiological mechanisms.

The potential associations between gut microbiota abundance and metabolites were explored using Spearman correlation analysis (Figure 5E). Further analysis revealed significant positive correlations between the abundance of wake‐related metabolites (cholesterol, acetylcholine, and nicotinic acid) and *Allobaculum*, *Enterococcus*, *Bacteroides* at the Phylum level (all *p* < 0.05). In contrast, these metabolites showed negative correlations with beneficial flora, including *Lactobacillales*, *Bifidobacterium, Muribaculaceae_genus*, *Romboutsia*, and *Allobaculum*. Notably, *Romboutsia* was specifically correlated with increased levels of adenosine and L‐Histidine (all *p* < 0.05).

## 4 Discussion

For the first time, we investigated the impacts of probiotic yogurt treatment on anesthesia‐induced sleep disturbances in rats. Using 16S rRNA gene amplicon sequencing analysis and untargeted metabolomic analysis, we compared the abundance and diversity of gut and lung microbiota and their metabolites in different groups and time points. Our study revealed that the diversity and abundance of gut microbiota in rats were significantly disrupted alongside post‐anesthesia sleep disturbances more than lung microbiota, with *Lactobacillales* and *Bifidobacterium* showing the most pronounced changes at the genus level. Subsequently, a targeted oral probiotic yogurt regimen demonstrated its substantial benefits in improving sleep quality following isoflurane anesthesia. This treatment not only increased the abundance of beneficial bacteria but also reduced wake‐related metabolites in both gut and lungs. These findings highlight that the targeted probiotic treatment may offer a promising dietary strategy for managing post‐anesthesia disturbances.

A growing body of research has revealed that the microbiota‐gut‐brain axis involves bidirectional communication between the CNS and the development and progression of sleep disorders (Wang, Wang, et al. 2022; Triplett et al. 2020). As a commonly used inhalational anesthetic, isoflurane works by altering the activity of neurotransmitters and ion channels in the brain, which may alter sleep architecture, affecting both the quality and structure of sleep (Mashour et al. 2010; Pick et al. 2011). In our study, we also found that isoflurane significantly impaired both sleep duration and sleep structure in rats. Additionally, the gut and lung microbiota in isoflurane‐anesthetized rats differed significantly from those in control rats, with a more pronounced impact on the gut microbiota compared to the lung microbiota. Previous studies have shown that the gut microbiota is highly sensitive to environmental and physiological changes. Isoflurane may affect the composition and function of the gut microbiota by altering gut blood flow, barrier function, and immune response (Serbanescu et al. 2019). Englert et al. (2015) reported that isoflurane reduced lung injury caused by sepsis and mechanical ventilation by protecting the integrity of the alveolar‐capillary barrier. However, further research is needed to explore the impact of anesthetic drugs on various organs and their associated microbiomes.

Evidence indicated that acute sleep deprivation decreased the gut abundance of *Lactobacillus*, *Akkermansia mucinphila*, and *Bifidobacterium*, as well as functional metabolites (Wang et al. 2022; Yang, Huang, et al. 2023), which is consistent with our study. *Lactobacillus* spp. and *Bifidobacterium* spp. have shown promising effects as food supplements for enhancing sleep and emerged as potential therapeutics. Lee et al. (2021) demonstrated that a probiotic mixture composed of *Lactobacillus reuteri* NK33 and *Bifidobacterium* significantly enhanced sleep quality in 156 individuals with symptoms of anxiety and insomnia. Supplementation with *Lactobacillus plantarum* JYLP‐326 could relieve insomnia symptoms in test anxious college students (Zhu et al. 2023). Hence, our study targeted bacterial groups sensitive to sleep disturbances for treatment to improve sleep inadequacy for health maintenance in post‐anesthesia rats. Remarkably, increasing beneficial floras contributed to sleep improvement, consistent with many similar studies (Lin et al. 2024; Chan et al. 2023).

Furthermore, increasing evidence indicates that microbiota metabolites and microbiota‐synthesized neurotransmitters can enter the CNS, modulating brain function and host behavior (Teichman et al. 2020). Among these metabolites and compounds promoting the wakefulness cycle (Holst and Landolt 2022; Haarhuis et al. 2022), acetylcholine and serotonin, along with nicotinic acid and cholesterol in the gut, and histamine and succinic acid semialdehyde in the lung, were significantly reduced in the probiotic group. In contrast, sleep‐promoting amino acids, such as γ‐aminobutyric acid (GABA), melatonin, adenosine, and L‐Histidine (Holst and Landolt 2022; Haarhuis et al. 2022), showed that only L‐Histidine was statistically increased in the lungs of probiotic‐treated rats compared to isoflurane group rats. L‐Histidine, an essential amino acid that serves as the sole precursor for histamine biosynthesis, has been proven to tightly regulate the sleep–wake cycle (Topriceanu et al. 2020). Emiko et al. (Aizawa et al. 2018) found a significant negative correlation between *Bifidobacterium* counts and cortisol levels associated with stress and sleep. Additionally, a cohort of 159 Koreans identified differential enrichment of *Bacteroides*, *Alloprevotella*, and *Prevotella_9* in individuals with poor sleep quality (Seong et al. 2024), which was linked to increased production of proinflammatory cytokines and the onset of various immune diseases (Zhou et al. 2022). These findings align with our microbiome‐metabolites correlation results.

However, we observed several differential metabolites associated with wakefulness and sleep in the lungs, with similar changes in the gut, despite limited alterations in the lung microbiome. Recent studies suggested that gut microbial communities release metabolites with multiple functions that transfer to various organs, including the lungs and brain (Anand and Mande 2018). Few studies have explored whether administration of differential metabolites from probiotics can replicate their sleep‐improving effects. Further research is needed to clarify the mechanisms underlying metabolite transport across different organs and facilitate the development of more targeted therapeutic agents.

Limitations of this study should be noted. First, no monitoring of anesthesia depth or physiological parameters was performed during isoflurane administration. Second, the mechanisms behind the improvement of sleep quality in rats by oral probiotic yogurt administration remain unclear. Third, our findings are based solely on an animal model, and the potential therapeutic effects of probiotics in post‐anesthesia patients have yet to be investigated.

## 5 Conclusion

Our study demonstrated that targeted probiotic treatment has beneficial effects on post‐anesthesia sleep disturbances in rats. Probiotic yogurt containing *Bifidobacterium animalis subsp*. and *Lactobacillus delbrueckii subsp*. significantly increased NREM sleep duration, reduced wakefulness, improved the gut microbiome, and modulated the production of sleep‐related metabolites in both gut and lungs. These findings suggest that targeted probiotic treatment holds significant potential as an effective therapeutic strategy for managing sleep disturbances in related patients.

## Author Contributions

**Rui‐zhi Yang:** conceptualization (equal), data curation (equal), formal analysis (equal), project administration (equal), writing – original draft (equal). **Song Lin:** conceptualization (equal), data curation (equal), formal analysis (equal), project administration (equal), writing – original draft (equal). **Le‐tong Huang:** formal analysis (equal), methodology (equal), project administration (equal), writing – original draft (equal). **Jing Weng:** conceptualization (equal), methodology (equal), project administration (equal), writing – original draft (equal). **Qiao‐ming Liu:** formal analysis (equal), methodology (equal), project administration (equal), writing – original draft (equal). **Han‐shen Chen:** formal analysis (equal), methodology (equal), project administration (equal), writing – original draft (equal). **Ning Ruan:** funding acquisition (equal), methodology (equal), supervision (equal), writing – review and editing (equal). **Kai Zeng:** funding acquisition (equal), methodology (equal), supervision (equal), writing – review and editing (equal).

## Funding

This study was supported by grants from the National Natural Science Foundation of China (82471491) to Z.K. and Fujian Provincial Financial Special Fund (2019B021) to R.N.

## Conflicts of Interest

The authors declare no conflicts of interest.

## Supporting information


**Figure S1:** Isoflurane anesthesia exerted little effect on the lung microbiome in rats. (A) Analysis of Alpha diversity (Shannon diversity index). (B) In the analysis of Beta diversity (PCoA), the aggregated distribution positions of each group indicate their similarity. (C) OTU Venn diagram showed that shared and differential OTUs were relatively low. (D) The absolute abundance of the lung microbiome in isoflurane‐anesthetized rats gradually decreased over time compared with the control group. (E) At the genus level, the absolute abundance of Bacteroidales increased after isoflurane inhalation, whereas Lactobacillales decreased, although there was no statistical difference. *n* = 5 for each timepoint.

## Data Availability

Data supporting the findings of this study are available from the corresponding author upon reasonable request.
