# Supplementary figures and images for: Effect of Targeted Probiotics on Anesthesia‐Induced Sleep Disturbances via Modulating the Gut Microbiome and Metabolites
Source: Food Sci Nutr. 2026 Jan 14;14(1):e71447. doi: 10.1002/fsn3.71447 (PMC12802074; doi:10.1002/fsn3.71447)

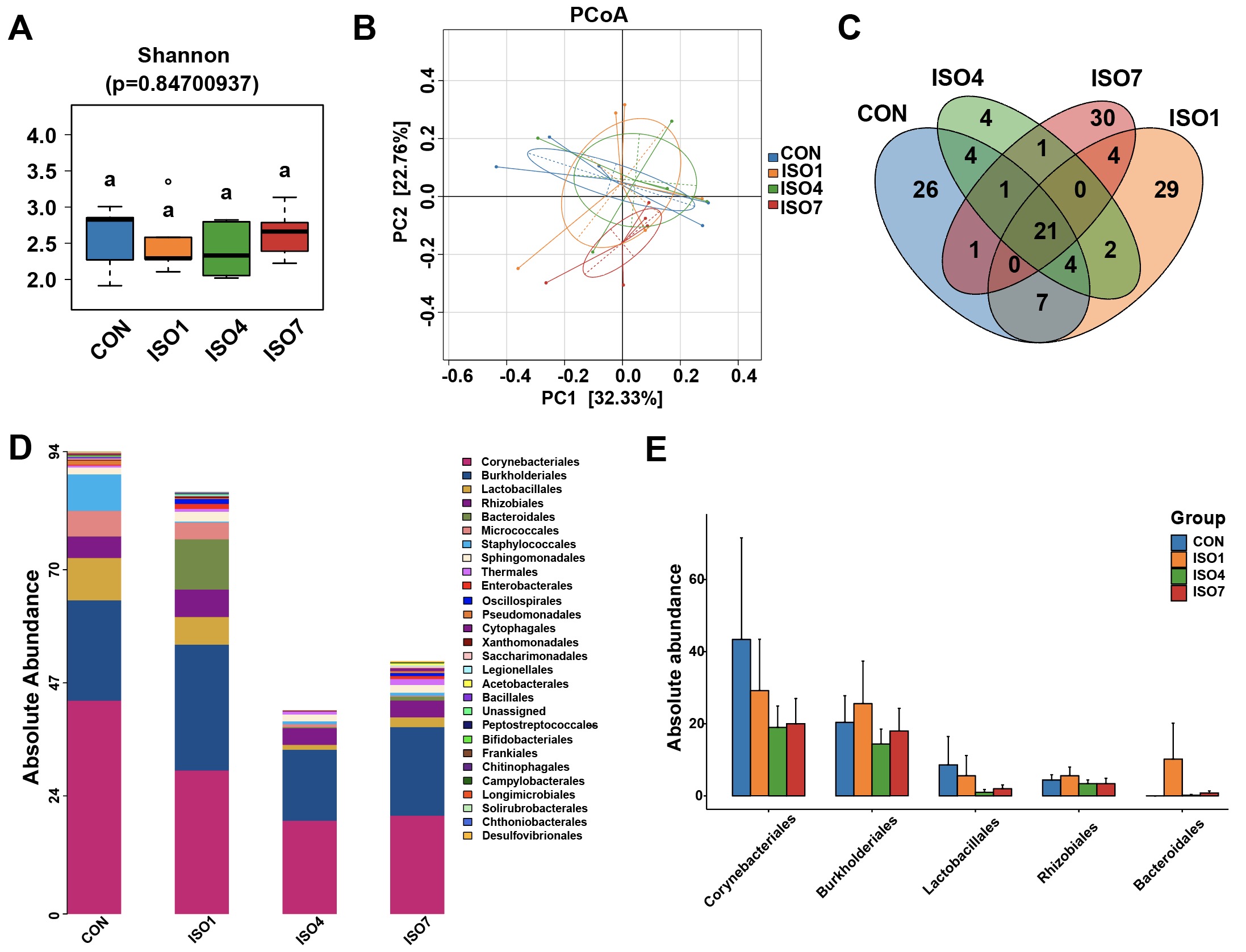

Supplement: Supplementary file 1 — Figure S1: Isoflurane anesthesia exerted little effect on the lung microbiome in rats. (A) Analysis of Alpha diversity (Shannon diversity index). (B) In the analysis of Beta diversity (PCoA), the aggregated distribution positions of each group indicate their similarity. (C) OTU Venn diagram showed that shared and differential OTUs were relatively low. (D) The absolute abundance of the lung microbiome in isoflurane‐anesthetized rats gradually decreased over time compared with the control group. (E) At the genus level, the absolute abundance of Bacteroidales increased after isoflurane inhalation, whereas Lactobacillales decreased, although there was no statistical difference. n = 5 for each timepoint. [file FSN3-14-e71447-s001.jpg]
